# Hepatitis C Virus Reflex Testing Protocol in an Emergency Department

**DOI:** 10.5811/westjem.2021.10.52468

**Published:** 2022-02-28

**Authors:** Jacob J. Manteuffel, Madison S. Lee, Rebecca M. Bussa, Noor L. Sabagha, Kaleem Chaudhry, Jacob E. Ross, Megan R. Cook, Jo-Ann K. Rammal, Karthik Sridasyam, Howard A. Klausner, Linoj P. Samuel, Joseph B. Miller

**Affiliations:** * Henry Ford Hospital, Department of Emergency Medicine, Detroit, Michigan; † Henry Ford Hospital, Department of Pathology, Detroit, Michigan

## Abstract

**Introduction:**

Our aim was to measure hepatitis C virus (HCV) screening and linkage-to-care rates in an urban emergency department (ED) before and after implementing an HCV viral RNA (vRNA) reflex testing protocol within a HCV screening program for at-risk patients. Our hypothesis was that using a reflex testing protocol would increase HCV testing rates of at-risk patients in the ED, which would increase the linkage-to-care rate.

**Methods:**

In August 2018, our institution implemented an automated, electronic health record-based HCV screening protocol in the ED for at-risk patients. In January 2019, we implemented an HCV vRNA reflex testing protocol (reflex testing) for all positive HCV antibody (Ab) tests that were initiated through the screening protocol. We compared completion rates of HCV vRNA testing and the rate of linkage to care for patients with positive HCV Ab test results before and after implementation of reflex testing (five months per study period).

**Results:**

Prior to reflex testing implementation, 233/425 (55%) patients with a positive HCV Ab test had an HCV vRNA test performed, whereas 270/323 (84%) patients with a positive HCV Ab test result had vRNA testing after reflex testing implementation (odds ratio [OR], 4.2; 95% confidence interval (CI): 3.0–6.0; P < 0.001). Of the eligible patients with positive HCV Ab test results who could be linked to care, 45 (10.6%) were linked to care before HCV reflex implementation and 46 (14.2%) were linked to care with reflex testing (OR, 1.4; 95% CI: 0.9–2.2; P = 0.13).

**Conclusion:**

Implementing a reflex testing initiative into an HCV screening program in the ED can result in an increase of the percentage of patients who receive an HCV vRNA test after having had a positive HCV Ab. Hepatitis C virus vRNA reflex testing was not associated with a statistically significant increase in linkage-to-care rates for HCV Ab-positive patients; however, further studies are required.

## INTRODUCTION

The hepatitis C virus (HCV) is a major cause of chronic liver disease and cirrhosis.^1^ In the United States, it is the most common chronic bloodborne pathogen, affecting almost 2% of the population.^2^ Globally, almost 71 million people have chronic HCV, with many people being unaware of their infection status^3^,^4^; almost 400,000 people worldwide die each year of complications from cirrhosis or hepatocellular carcinomas caused by HCV.^4^ Reports estimate that between 1–12% of eligible adults are tested for HCV,^5^ and if current rates of identification remain constant, the estimated HCV-related morbidity and mortality could quadruple over the next decade.^3^ Thus, the World Health Organization (WHO) has established a goal to reduce new viral hepatitis infections by 90% and reduce deaths due to HCV by 65% by 2030.^4^ To achieve these goals, expanding and improving HCV screening processes will be critical.

The most common route of HCV transmission has historically been through blood transfusions.^6^ However, blood donations are now regularly screened for HCV and donors are asked questions about HCV risk factors. Currently, the most common HCV transmission route in high-income countries is intravenous (IV) drug use.^7^ Other routes of HCV transmission occur less frequently.^7^ For example, transmission from an infected mother to a child occurs in about 5–10% of pregnancies and is more likely to occur when women are infected with human immunodeficiency virus (HIV).^6^ Hepatitis C virus can also be transmitted through workplace needle-stick injuries^6^,^7^ and can be sexually transmitted. Additionally, individuals receiving tattoos, those infected with HIV, and men who have sex with men are considered at-risk groups.

Prior to April 2020, the US Centers for Disease Control and Prevention (CDC) recommended HCV testing for current or former IV drug users, those born between 1945–1965, recipients of blood transfusions before 1992, healthcare workers who had work-related needle sticks with blood products, people with HIV, and children born to mothers with HCV. In 2020, the recommendations were augmented to include testing for pregnant women and at least one lifetime HCV test for adults 18 years and older.^8^ The American Association for the Study of Liver Diseases recommends annual HCV screening for IV drug users and men who have sex with men.^1^

The Frontlines of Communities in the United States (FOCUS) program is a quality improvement (QI) effort that was created in 2010 to tackle the HIV epidemic.^9^ It was designed to align screening practices in the emergency department (ED) with CDC guidelines and to promote linkage-to-care practices in the ED. In light of the recommendations from the CDC and the stated goals of the WHO to significantly reduce new HCV infections, FOCUS was expanded in 2014 to develop CDC-compliant HCV screening, diagnosis, and linkage-to-care practices. Linkage to care is the process by which patients infected with HCV are identified through virus screening, notified of their HCV diagnosis, and “linked” to healthcare providers who can offer HCV treatment and care.

**Population Health Research Capsule**

What do we already know about this issue?

*The emergency department (ED) patient population in the United States has a relatively high burden of undiagnosed hepatitis C virus (HCV) infection.*

What was the research question?

*Does viral RNA (vRNA) reflex testing within an HCV screening program improve screening and linkage-to-care rates in the ED?*

What was the major finding of the study?

*The percentage of ED patients who received an HCV vRNA test after having had a positive HCV antibody test result increased.*

How does this improve population health?

*Screening for HCV with reflex vRNA testing in the ED has the potential to identify and treat patients with HCV and decrease the morbidity and mortality related to infection.*

Diagnosing HCV is a two-step process. First, an anti-HCV antibody (Ab) test is performed, which can reveal an active infection or a previous infection that has resolved.^6^ The most common anti-HCV Ab test is an enzyme-linked immunosorbent assay,^6^ which has 95% sensitivity and specificity and can detect Ab from 4–10 weeks after infection.^6^ After a positive Ab test, an HCV viral RNA (vRNA) polymerase chain reaction assay assesses whether the infection is active and determines viral load.^1^,^6^ To ensure complete and timely diagnosis of HCV for at-risk patients, reflex testing is recommended. In laboratory reflex testing, positive HCV Ab test results trigger an automatic vRNA test on the same patient sample, provided that the sample volume is sufficient.^10^ Studies of ED HCV screening programs have referenced reflex testing,^11^ and others have reported that reflex testing leads to a high percentage of positive HCV Ab tests with a reflexive vRNA test result.^12^ However, to our knowledge, no studies have examined whether implementation of HCV reflex testing improves institutional HCV screening rates and linkage-to-care program utilization.

Our aim was to measure HCV screening and successful linkage-to-care rates in an urban emergency department (ED) before and after implementing an HCV vRNA reflex testing protocol within an HCV screening program for at-risk patients. Our hypothesis was that using a reflex testing protocol would increase HCV testing rates of at-risk patients in the ED, which would increase the linkage-to-care rate. Ultimately, robust HCV screening and efficient linkage to care are needed to improve patient quality of life and lower HCV transmission. Our single-institution, QI pilot study was performed to establish proof of principle for HCV reflex testing utility in improving HCV screening and linkage to care in the ED.

## METHODS

### Study Design and Setting

This QI study was approved by the Henry Ford Hospital Institutional Review Board, and the requirement for informed consent was waived. Annual ED patient volume at our institution is approximately 100,000. In August 2018, our institution implemented an automated electronic health record (EHR)-based HCV screening protocol in the ED for at-risk patients. In January 2019, we implemented a HCV vRNA reflex testing protocol (reflex testing) for all positive HCV Ab tests that were initiated through the screening protocol. We compared completion rates of HCV vRNA testing and the rate of linkage to care for patients with positive HCV Ab test results before and after implementation of reflex testing (five months per study period).

### Study Protocol

The EHR-based HCV screening protocol implemented in August 2018 was designed for minimal disruption to the ED workflow, so as not to prolong ED length of stay. A previous study^13^ has suggested that limiting HCV screening to patients for whom emergency clinicians already intend to order a blood test performed would not increase ED length of stay. This study suggests that HCV screening can be linked to frequently and commonly ordered tests (such as a complete blood count) to minimally impact nursing workflow. Our protocol automates HCV screening when an emergency clinician orders a complete blood count in the institution’s EHR system Epic (Epic Systems, Verona, WI), which verifies whether the patient was born between 1945–1965 and whether the patient has a history of IV drug use. These criteria were selected based on CDC HCV screening recommendations. We also customized Epic to exclude this order if patients had previously completed a test for HCV Ab or had a history of HCV.

**Figure 1:**
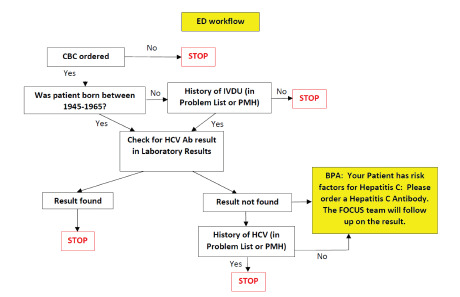
Electronic health record algorithm when a clinician in the emergency department orders a complete blood count. *ED*, emergency department, *CBC*, compete blood count; *Ab*, antibody; *BPA*, best practice advisory; *HCV*, hepatitis C virus; *IVDU*, intravenous drug use; *PMH*, past medical history.

**Figure 2:**
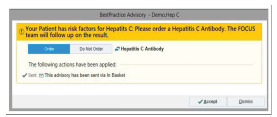
Example of the best practice advisory notice that appears when a complete blood count is ordered. *FOCUS*, Frontlines of Communities in the United States; *Hep*, hepatitis.

For patients eligible for automated ordering, a best practice advisory (BPA) alert suggests that the patient qualifies for HCV Ab testing prior to a clinician signing the order (Figure 1). The BPA is a box that appears on the emergency clinician’s Epic screen with text stating that the patient has “Risk Factors for Hepatitis C. Please order a Hepatitis C Antibody. The FOCUS team will follow up on the result.” The BPA is pre-populated by default to “order” the test; however, the option “do not order” is provided, giving the clinician flexibility to accept or dismiss the order (Figure 2). If the order is accepted, it is added to the list of orders for the clinician to sign. Patients were given the option to decline testing prior to blood draw. Emergency clinicians were not required to wait for test results before admitting or discharging the patient so as not to prolong ED length of stay. The clinicians were educated on how to notify patients of positive HCV Ab tests and were directed to emphasize that positive Ab screening tests require confirmatory HCV vRNA testing for diagnosis.

As part of the QI project, we received a weekly Epic-generated report of the preceding week’s HCV test results. Positive Ab tests were identified in this report, and we contacted patients to return for further HCV vRNA testing and linkage to outpatient HCV care as needed. In January 2019, our institution implemented HCV vRNA reflex testing to the HCV screening program. During reflex testing, serum from blood samples that had tested positive for HCV Ab were automatically sent for HCV vRNA testing. The laboratory validated serum as an alternative specimen type and performed testing to determine that there was no risk of cross-contamination in using the same sample for both HCV Ab and HCV vRNA. The HCV Ab testing was performed on a Siemens Centaur (Siemens Healthcare, Malvern, PA) instrument, and HCV vRNA reflex testing was performed using the Roche Cobas Ampliprep/Taqman HCV vRNA assay (Roche Diagnostics, Indianapolis, IN).

We quantified the staff time effort that was needed to link patients to care and compared effort time before and after HCV reflex testing implementation. To do this, we broke down the process of follow-up and linkage to care into eight steps and had team members perform time estimates for each step. If a patient attended their appointment, they were designated as “linked to care.” After three attempts to contact a patient via telephone with no response, the patient was designated as lost to follow-up. Using these times, we estimated the time needed to link patients to care per patient before and after the implementation of HCV vRNA reflex testing.

We performed a chart review of patients deemed linked to care to determine whether the patient followed up in our health system. For patients following up in our health system we determined whether the patient was treated and cured of HCV, indicated by a negative HCV vRNA test result after initiation of treatment for HCV.

### Data Analysis

Analysis consisted of descriptive statistics and univariate comparisons. For the primary outcome of analysis, we used chi-square analysis to compare the proportion of HCV Ab-positive patients linked to care before and after HCV vRNA reflex testing. We present unadjusted odds ratios (OR) with corresponding 95% confidence intervals (CI). Analysis was performed with SAS 9.4 (SAS Institute, Inc., Cary, NC).

## RESULTS

From August 1, 2018–May 31, 2019, a total of 10,812 patients were screened with the BPA-initiated HCV screening protocol, triggering 6702 completed HCV Ab tests (62%). A total of 4077 of 7080 (58%) eligible patients were tested for HCV Ab before and 2625 of 3732 (70%) eligible patients were tested for HCV Ab after reflex testing was begun (Table 1). The rate at which clinicians in the ED accepted the BPA suggestion to order the HCV Ab test increased from 58% in the five-month period before reflex testing was started to 70% in the five-month period after reflex testing was begun. Prior to reflex testing implementation, 233/425 (55%) patients with a positive HCV Ab test had an HCV vRNA test performed, whereas 270/323 (84%) patients with a positive HCV Ab test result had vRNA testing after reflex testing implementation (OR, 4.2; 95% CI, 3.0–6.0; *P <*0.001).

For all patients included in this analysis, the HCV Ab positivity rate was 11.2% (748/6702), whereas the HCV vRNA positivity rate was 62.6% (315/503). Rates of positive HCV Ab and HCV vRNA were similar before and after reflex testing implementation (Table 1). Of the potentially eligible patients with positive HCV Ab test results who could be linked to care, 45 (10.6%) were linked to care before HCV reflex implementation and 46 (14.2%) were linked to care with reflex testing (OR, 1.4; 95% CI, 0.9–2.2; *P* = 0.130). Of the 91 patients linked to care, 58 patients were linked to care within our health system. Of those 58 patients linked to care in our health system, 22 (38%) patients had negative HCV vRNA testing after initiation of HCV treatment indicating cure of disease. Team member estimates of the time spent coordinating linkage to care prior to reflex testing was 51 minutes per patient before reflex testing compared to 28 minutes per patient after reflex testing was initiated. The time saved was largely due to eliminating steps required to contact patients and have them return to the laboratory for HCV vRNA testing prior to linkage to care.

## DISCUSSION

In this study, we observed that after implementing a reflex testing initiative into an HCV screening program in the ED, the percentage of patients who received an HCV vRNA test after having had a positive HCV Ab test result significantly increased. We also saw that HCV vRNA reflex testing was associated with a numerical increase in linkage to care for HCV Ab-positive patients; however, these results did not reach statistical significance. Additionally, we saw that the team member time spent to link patients to care was decreased when reflex testing was used. Given that HCV vRNA test results were positive for 62.6% of our patients who had a positive HCV Ab test result, our rate of linking 14.2% of HCV Ab-positive patients to care after implementing reflex testing represents just under one quarter of patients who could potentially have been linked to care. The importance and value of HCV vRNA reflex testing with an HCV screening program is yet to be described in the literature, and our pilot quality improvement study suggests that it may be a good strategy for improving HCV surveillance and care.

In the United States, there is a paucity of published literature addressing the importance and efficacy of HCV vRNA reflex testing. Studies in Spain have reported an increased prevalence of HCV vRNA reflex testing in Spanish hospitals after specialty societies advocated for reflex testing and improvement of patient linkage to care after implementation of reflex testing.^14^,^15^ Reflex testing has been noted to promote a high percentage of HCV vRNA tests for patients with positive HCV Ab tests in an ED HCV screening program; however, the relative increase in HCV vRNA tests done, the impact on linkage to care, and quantification of time saved were not quantified.^16^ The high percentage of HCV vRNA tests done in that study (approaching 98%) is a testament to the thoughtfulness and pre-planning undertaken prior to the implementation of their HCV screening program.

Over our 10-month study period, the BPA-initiated, EHR-based HCV screening program showed an increase in the rate at which the emergency clinicians accepted the BPA suggestion to order an HCV Ab test. Our overall BPA acceptance rate of 62% compares favorably to another study that measured this metric.^16^ This is likely the result of increased clinician familiarity and confidence with the HCV screening program. Because some clinicians may be uncomfortable ordering tests that are not directly relevant to the patient’s presenting concern, ongoing education regarding the HCV screening program among residents, staff physicians, and mid-level healthcare staff is important for the success of the program. In addition, follow-up and sharing of patient success stories with healthcare clinicians and nursing staff are critical to a successful program.

In our study, fewer HCV Ab tests were performed after the start of reflex testing than were done before reflex testing, which might be explained by a smaller cohort of eligible patients after program initiation; however, despite the lower number of tests done, a higher percentage of patients with a positive Ab test had vRNA testing performed when reflex testing was active. The screening program was designed so that after a patient has had HCV Ab testing in their record, a BPA will no longer prompt the emergency clinician to order testing. We now intend to expand the HCV screening protocol to correspond to new CDC guidelines, which recommend screening all patients 18 years and older at least once in a lifetime. For those at higher risk, such as patients who use IV drugs, we will program the EHR algorithm to prompt for a new test annually, even if the patient has a negative HCV Ab test result in the health record.

While HCV vRNA reflex testing is designed to be implemented for 100% of HCV Ab-positive samples, our results showed an 84% completion rate. This was almost entirely due to inadequate volume of the initial sample as indicated by the laboratory comment “quantity not sufficient,” which suggests that initial testing protocols can be improved to reduce technical problems in the testing pipeline. The HCV Ab test requires 50 microliters (μL) of serum while HCV vRNA testing requires a minimum of 700 μL of serum or plasma. A 2014 study using a simulation model to assess interventions that might improve HCV treatment rates suggested the need for interventions that focus on multiple points within the trajectory of care,^17^ and this observation highlights the need for attention to even small details such as sample acquisition.

Our institution strives to increase the percentage of HCV patients linked to care and decrease the number of patients lost to follow-up, but a range of issues makes this challenging. Restricting the eligible clinicians who treat HCV (limited to hepatology and infectious disease) can create extended appointment wait times and limit efforts at establishing linkage to care. Also, our patient population generally has limited access to certain resources, such as a lack of communication devices, transportation, and social support, as well as having concomitant poverty and substance use disorders, which could hinder linkage-to-care efforts. Sobriety restrictions on treatment medications also hinder the overall goal of eliminating HCV. Current efforts to overcome our challenges in linkage to care include a text message campaign to contact patients and the development of a real-time, EHR-based notification process to notify the project team when patients previously deemed lost to follow-up register to be seen in our ED. Telehealth and e-visits could also potentially increase the likelihood that patients can be linked to care. These efforts, combined with increased collaboration with our local and state health departments, are underway to improve our linkage-to-care rates.

While our time estimates for linkage to care from the team member survey are admittedly rudimentary, estimating the amount of time needed for team member staff to successfully implement and operate an HCV screening program is critical to future efforts to eliminate HCV. Additionally, given our time estimates, an HCV vRNA reflex component added to an HCV screening program may cut necessary staff time by nearly half and, therefore, should be considered an essential component to implementation of a program. Our time calculations represented the minimal amount of time needed to follow the linkage-to-care process and did not address the multitude of challenges that come with this process. Challenges include patient transportation issues, missed appointments, language barriers, and often multiple telephone call attempts needed for each step in the process.

With respect to cost, our institution charges $7 for each HCV Ab test and $120 for each HCV vRNA test. We performed 6702 HCV Ab tests and 503 HCV vRNA tests in this analysis with total laboratory charges of $107,274. Of the 91 patients linked to care, 58 had follow-up in our health system allowing for further chart review and 22 (38%) of those had negative HCV vRNA testing after HCV treatment, indicating cure of disease. Extrapolation of that percentage to all patients linked to care would yield approximately 35 patients cured of disease. Therefore, the cost of HCV screening would be approximately $1179 per patient linked to care and $3064 per patient cured of disease. It is important to note that private and public insurers typically pay a fraction of charged costs; thus, true financial costs are likely lower than these estimates. Differences in prevalence of disease in other settings would impact these estimates.

Reimbursement for the services of healthcare navigators might be another cost-effective measure to create a self-sustaining HCV screening program and make strides in efforts to eliminate HCV. The long-term goal for our HCV screening program is to create a self-sustaining program that aids the common goal of eliminating HCV. Implementing HCV vRNA reflex testing could significantly decrease cost, time, and effort needed to reach the goal of self-sustainability.

## LIMITATIONS

There are several limitations to this study that are inherent to the nature of public health qualitive improvement initiatives. Study members were not blinded to outcomes, which may have generated bias in the linkage-to-care rate. It is also possible that a small percentage of the patients in the cohort after HCV vRNA reflex implementation had their HCV vRNA tests run using the pre-implementation algorithm, as their samples may have returned as “quantity not sufficient.” Reliable contact information for patients was also a limitation and may have resulted in incomplete data collection. The methods used to estimate the time needed to link the patients to care may not be generalizable and may vary by healthcare clinician, depending on ability and level of training. Due to limitations of data collection methods, we were unable to obtain the statistics to determine the percentage of patients the BPA fired for due to age cohort criteria vs IV drug use criteria.

## CONCLUSION

Implementing a reflex testing initiative into an HCV screening program in the ED can result in an increase of the percentage of patients who receive an HCV vRNA test after having had a positive HCV Ab. Therefore, reflex testing should be considered as part of an ED-based HCV screening program. The HCV vRNA reflex testing was not associated with a statistically significant increase in linkage-to-care rates for HCV Ab-positive patients; however, further studies are required.

Preliminary results from this study were accepted for presentation at the Society of Academic Emergency Medicine 2020 Virtual Meeting, May 12–15, 2020.

## Figures and Tables

**Table 1:** Hepatitis C virus screening and linkage to care before and after initiation of reflex testing. ^*^P < 0.001, chi-square test comparing viral RNA tests completed pre- and post-reflex testing.

	Study phase		

	Pre-reflex testing August 1, 2018–December 31, 2018 No. (%)	Post-reflex testing January 1, 2019–May 31, 2019 No. (%)	Entire study period August 1, 2018–May 31, 2019 No. (%)
HCV Ab BPA fires	7,080	3,732	10,812
HCV Ab BPA triggered orders	4,077 (58)	2,625 (70)	6,702 (62)
HCV Ab tests positive (% of orders triggered)	425 (10.4)	323 (12.3)	748 (11.2)
HCV vRNA tests completed (% of those ordered)	233 (55)	^*^270 (87)	503 (67)
HCV vRNA tests positive (% of those completed)	145 (62.2)	170 (63)	315 (63)
Patients linked-to-care (% of positive Ab tests)	45 (10.6)	46 (14.2)	91 (12.2)

*Ab*, antibody test; *BPA*, best practices advisory; *HCV*, hepatitis C virus; *vRNA*, viral RNA.

## References

[1] Wilkins T, Akhtar M, Gititu E (2015). Diagnosis and management of hepatitis C. Am Fam Physician.

[2] Owens DK, Davidson KW, U S. Preventive Services Task Force (2020). Screening for hepatitis C virus infection in adolescents and adults: US Preventive Services Task Force Recommendation Statement. JAMA.

[3] Sidlow R, Msaouel P (2015). Improving hepatitis C virus screening rates in primary care: a targeted intervention using the electronic health record. J Healthc Qual.

[4] World Health Organization (2020). Hepatitis C fact sheet.

[5] Linas BP, Hu H, Barter DM (2014). Hepatitis C screening trends in a large integrated health system. Am J Med.

[6] Mehta N, Carey W, Alkhouri N (2017). Hepatitis C.

[7] Shepard CW, Finelli L, Alter MJ (2005). Global epidemiology of hepatitis C virus infection. Lancet Infect Dis.

[8] Schillie S, Wester C, Osborne M (2020). CDC recommendations for hepatitis C screening among adults - United States, 2020. MMWR Recomm Rep.

[9] Sanchez TH, Sullivan PS, Rothman RE (2014). A novel approach to realizing routine HIV screening and enhancing linkage to care in the United States: protocol of the FOCUS Program and early results. JMIR Res Protoc.

[10] Ryerson AB, Schillie S, Barker LK (2020). Vital Signs: Newly reported acute and chronic hepatitis C cases - United States, 2009–2018. MMWR Morb Mortal Wkly Rep.

[11] Blackwell JA, Rodgers JB, Franco RA (2020). Predictors of linkage to care for a nontargeted emergency department hepatitis C screening program. Am J Emerg Med.

[12] Calner P, Sperring H, Ruiz-Mercado G (2019). HCV screening, linkage to care, and treatment patterns at different sites across one academic medical center. PLoS One.

[13] White DA, Anderson ES, Pfeil SK (2016). Hepatitis C virus screening and emergency department length of stay. PLoS One.

[14] Crespo J, Lazaro P, Blasco AJ (2021). Hepatitis C reflex testing in Spain in 2019: a story of success. Enferm Infecc Microbiol Clin.

[15] Casas MP, García F, Freyre-Carrillo C (2020). Towards the elimination of hepatitis C: implementation of reflex testing in Andalusia. Rev Esp Enferm Dig.

[16] Schechter-Perkins EM, Miller NS, Hall J (2018). Implementation and preliminary results of an emergency department nontargeted, opt-out hepatitis C virus screening program. Acad Emerg Med.

[17] Linas BP, Barter DM, Leff JA (2014). The hepatitis C cascade of care: identifying priorities to improve clinical outcomes. PLoS One.

